# Importance of Targeted Communication Strategies During COVID-19 Vaccination Campaigns in Mozambique: Results of a Mixed-Methods Acceptability Study

**DOI:** 10.1093/cid/ciaf054

**Published:** 2025-07-22

**Authors:** Caroline De Schacht, Igor Ubisse Capitine, Carlota Lucas, Ana Muteerwa, Paula Paulo, Alvaro Manhiça, Fernando Padama, Celso Belo, Leonildo Nhampossa, Graça Matsinhe, Zhihong Yu, C William Wester

**Affiliations:** Centro Pela Saúde Global (C-Saúde), Evaluations Department, Maputo, Mozambique; Instituto Nacional de Saúde, Maputo, Mozambique; Centro Pela Saúde Global (C-Saúde), Evaluations Department, Maputo, Mozambique; US Centers for Disease Control and Prevention, Division of Global HIV and TB, Maputo, Mozambique; Centro Pela Saúde Global (C-Saúde), Evaluations Department, Maputo, Mozambique; Instituto Nacional de Saúde, Maputo, Mozambique; Provincial Health Directorate of Zambézia, Operations Research Unit, Quelimane, Mozambique; Centro Pela Saúde Global (C-Saúde), Evaluations Department, Maputo, Mozambique; Ministry of Health, National Directorate of Public Health, Maputo, Mozambique; Ministry of Health, National Directorate of Public Health, Maputo, Mozambique; Vanderbilt University Medical Center (VUMC), Department of Biostatistics, Nashville, Tennessee, USA; Vanderbilt University Medical Center (VUMC), Vanderbilt Institute for Global Health (VIGH), Nashville, Tennessee, USA; Vanderbilt University Medical Center (VUMC), Department of Medicine, Division of Infectious Diseases, Nashville, Tennessee, USA

**Keywords:** COVID-19 vaccination, acceptability, Mozambique, communication strategies

## Abstract

**Background:**

Mozambique implemented a phased roll-out of COVID-19 vaccination in 2021. This study aimed to evaluate COVID-19 vaccine acceptability among higher-risk populations in Zambézia Province.

**Methods:**

A mixed-methods study in Zambézia Province assessed knowledge, perceptions, and acceptability of COVID-19 vaccination. Structured questionnaire-based surveys among community health workers/volunteers, taxi drivers, and persons with HIV; and focus group discussions using semi-structured guides with community/religious leaders, adults aged 18–49 years and adults aged 50+ years were conducted in August–September 2021. Surveys were captured using tablets; group discussions were recorded. Univariate analyses were performed for quantitative data; qualitative data were analyzed thematically.

**Results:**

A total of 731 individuals participated (539 survey respondents; 192 discussion respondents); 53% male (n = 386) and 74% urban (n = 544) residents. Most had heard about COVID-19 vaccines, mainly through television and/or radio. Trustworthy information sources mentioned were community leaders and healthcare providers. Among survey respondents, 249/539 (46%) reported having received at least 1 vaccine dose. Motivators for vaccination mentioned by survey respondents were “belief that vaccines protect themselves” (72%), “belief it would protect their family” (17%). Myths and beliefs, misinformation, and long queues were main barriers for vaccination mentioned in group discussions. Participants suggested that campaigns should focus on communication talks led by health professionals, in partnership with community or church leaders and/or community health workers/volunteers.

**Conclusions:**

This study showed that information on COVID-19 vaccination had successfully reached the vast majority of study participants, mainly via radio and television. Targeted campaigns specific for rural and urban contexts can increase awareness and uptake of vaccination.

## Teaser

This mixed-methods study conducted in Mozambique showed that information regarding COVID-19 vaccination reached the population. About half accepted vaccination, where myths, misinformation, wait time were seen as barriers. Targeted campaigns adapted for different contexts can increase awareness and vaccination uptake.

The Coronavirus Disease 2019 (COVID-19) is a significant global public health crisis that has overwhelmed healthcare systems, resulting in a high number of illnesses and deaths [1–5]. Despite unprecedented efforts and collaboration to produce and provide access to vaccines against COVID-19 [6], their access and distribution has challenged countries with limited resources and experience in vaccinating adult populations, such as Mozambique [7, 8].

In Mozambique, as in many low-income countries, the successful execution of the vaccination program relied on rapidly acquiring vaccine stocks and establishing a robust COVID-19 surveillance system. The country began receiving vaccines in March 2021 through COVID-19 Vaccines Global Access (the COVAX) initiative [9] and other mechanisms/ donations, initially on an irregular basis. The national plan included the crucial step of prioritizing specific population groups (healthcare workers, elderly individuals, people with chronic diseases as first target groups) for vaccination, until all of the defined target groups, and finally the general population, were covered (Supplementary Table 1) [10, 11].

Vaccine hesitancy, defined as “the delay in acceptance or refusal of vaccination even if vaccines and services are available” [12], consequently contributes to the problem of low vaccination coverage and a risk of not achieving the desired effect of population immunity or herd immunity [13]. Factors associated with vaccine hesitancy are time- and context-specific [14, 15]. A study conducted among adults living in sub-Saharan African (SSA) countries and those living in the diaspora found that knowledge and perceptions of COVID-19 vaccines, levels of education, and history of previous vaccinations influenced resistance and hesitancy [16]. Another review, which included 71 studies conducted in 17 African countries, reported that significant reasons for vaccine hesitancy were participants’ concerns about vaccine safety and side effects and misinformation or conflicting information from the media [14].

Mozambique is one of the few low-income countries reaching a high COVID-19 vaccination coverage (77% of the eligible population being fully vaccinated), one of the highest on the African continent [17]. Despite this initial success, it is crucial that monitoring of coverage among different groups and contexts is done to identify successes and challenges, and to be able to improve vaccination campaigns and maintain high coverage. This study aimed to evaluate COVID-19 vaccine acceptability in populations at greater risk of severe infection in Zambézia Province, Mozambique. Understanding which factors influence vaccine uptake can help in the development of personalized and contextualized interventions in order to promote optimal vaccine acceptability and success of future vaccination campaigns targeting adults.

## METHODS

### Study Setting

**Figure 1.**
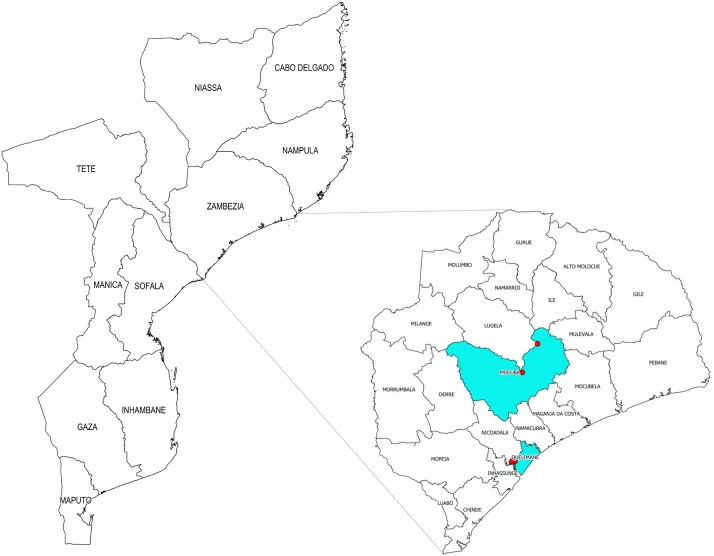
Study locations in the 2 selected districts (marked with red dots). Figure by C-Saúde.

The study was conducted in Zambézia, a predominantly rural province in central Mozambique, with an estimated population of 5.5 million [18]. Our study was implemented in 4 locations of 2 districts, Quelimane City District (urban) and Mocuba District (rural) (Figure 1). In Quelimane City, 2 neighborhoods with a higher seroprevalence of severe acute respiratory syndrome coronavirus 2 (SARS-CoV-2) antibodies were selected [19]. In the rural district, the locations of Mocuba and Mugeba were selected because of their rural location, having a large population, and being located on the main road (i.e., transit corridor) that connects south to north in the country.

### Study Design and Study Population

A cross-sectional, mixed-methods design was used. The study population included 6 different target groups: community health workers (CHWs)/volunteers, bicycle/motorcycle taxi drivers, persons with human immunodeficiency virus (PWH) attending any human immunodeficiency virus (HIV) services at the health facility, community/faith leaders, and community members (persons 18–49 years of age and 50+ years of age). Residents of Zambézia Province typically rely heavily on bicycle/motorcycle taxi services for their transportation needs; therefore, taxi drivers were identified by local partners as a group at higher risk of SARS-CoV-2 exposure because of frequent contact with the population. A SARS-CoV-2 survey in Quelimane showed a higher prevalence among health providers and taxi services and thus identified as higher risk [20]. General inclusion criteria (for all study population groups) were as follows: being an adult (18 years of age or older), being able to provide informed verbal consent, and being based in the city, community, or health facility selected for the evaluation. Specific inclusion and exclusion criteria are described in Table 1.

**Table 1.** Inclusion and Exclusion Criteria, per Target Group

Participant Group	Inclusion Criteria	Exclusion Criteria
Community health workers (CHWs) and volunteers (mentor mothers and peer educators)	18 years of age or older Being either a community health volunteer, such as a peer educator or mentor mother; doing the work of connecting with the community and selected HFs; or Being a CHW in either of the selected districts	Allocated to the selected HF less than 3 months from the beginning of data collection
Bicycle/motorcycle taxi drivers	18 years of age or older Individuals working as bicycle or motorcycle taxi drivers in Quelimane City or Mocuba Districts	N/A
Persons with HIV (PWH) attending HIV/ART services	18 years of age or older Attending HIV-related services on the day of data collection (either a new patient or patient in follow-up)	N/A
Community/religious leaders	18 years of age or older Being a community/faith leader and resident in one of the neighborhoods surrounding the HF selected for the study	N/A
Adult population from the community	18–49 years of age Individual of the community living in one of the neighborhoods surrounding the HF selected for the study	N/A
Older-aged adult population from the community	50 years of age or older Individual in the community living in one of the neighborhoods surrounding the HF selected for the study	N/A

Abbreviations: ART, antiretroviral therapy; HF, health facility; HIV, human immunodeficiency virus; N/A, not applicable.

### Study Procedures

Data were collected from August 2021 to September 2021. Before data collection, information sessions aimed at the respective groups were held at health facilities for CHWs/volunteers and PWH, taxi driver associations for taxi drivers, and neighborhood settings for community/faith leaders and community members. After the information sessions, individuals available and interested in participating were referred to the study team for further information and eligibility assessment and to obtain informed consent (if eligible). For focus group discussions (FGDs), location and time of the discussion were coordinated for all interested eligible individuals, while the informed-consent process was held just before the discussion.

A structured instrument was used for surveys and a semi-structured guide for the FGDs. Topics on COVID-19 vaccine knowledge, attitudes regarding COVID-19 vaccination, acceptance, and barriers to and facilitators/motivators for acceptance were explored.

COVID-19 prevention measures were followed throughout the period of data collection: each evaluation team member used personal protective equipment; all participants received a facemask; verbal consent was obtained from all participants to avoid contact through handling pens and papers; and all consent processes, surveys, and FGDs were held outdoors or in ventilated rooms, maintaining a minimum distance of 1.5 meter while securing confidentiality and privacy during the activities.

### Statistical Considerations

The sample size for the qualitative components was based on existing literature for qualitative research regarding hesitancy for COVID-19 vaccination [21], and hesitancy for other vaccines [22, 23], as only a few publications specifically on COVID-19 vaccine hesitancy using qualitative methods were available at the time of protocol writing. The final sample size for qualitative component was determined by saturation of content, as per qualitative methodology [24, 25], estimating 1–2 FGDs per study site. For the quantitative method (survey), we hypothesized an acceptability of 75%, based on existing data in African countries [26]. With a confidence interval (CI) of 95%, we estimated that at least 47 participants from each target group and per site (CHWs/volunteers, taxi drivers, and PWH) would be sufficient to estimate the underlying acceptability, assuming an absolute margin of error of 12.5%.

### Ethical Considerations

The study protocol and instruments were reviewed and approved by the local ethics committee (Institutional Health Ethics Committee of Zambézia [CIBS-Z], reference 22/CIBS-Z/21) and the Vanderbilt University Medical Center Institutional Review Board (#201887) [27, 28]. All participants provided verbal informed consent prior to data collection.

### Data Analysis

Descriptive analysis was conducted for the surveys through frequency tables and cross-tabulations; continuous variables were presented by means, medians, and interquartile intervals. A multivariable generalized linear mixed-effects model (GLMM) was used to assess how vaccine acceptance (defined by having received at least 1 dose) varies with age, sex, and target groups (PWH, CHWs/volunteers, and taxi drivers). Study locations were treated as random effects. Three missing values for age were imputed using the mean age when the GLMM was built. The R statistical software was used for the quantitative analysis [29].

Records and notes of the FGDs were transcribed verbatim. An initial codebook was developed based on existing literature and according to the study objectives and was refined after hearing/reading a representative number of FGD transcripts. Coding was independently done by 2 teams of 2 investigators (4 coders in total); all coding that was agreed upon between the investigators was considered for analysis. Thematic analysis was conducted to code and group the main themes identified by reading the transcripts [30]. Qualitative analysis was supported by MAXQDA 2018 (VERBI Software).

## RESULTS

### Participant Characteristics

In this study, 731 adult individuals from diverse population groups participated, with 74% (539/731) being survey participants composed of CHWs/volunteers, PWH attending HIV services, and taxi drivers; 26% (192/731) were community members participating in FGDs, including community/faith leaders and adult members of the community. Nearly three-quarters of all participants were from urban areas. Approximately one-quarter (169/731) of participants were under the age of 25, and 26% (192/731) had not received a formal education. With regard to mother language, 83% (603/731) reported a local language, and only 17% had Portuguese as first language (Table 2).

**Table 2.** Sociodemographic Characteristics of the Study Participants

	Total (n = 731)	Survey Respondents (n = 539)	FGD Respondents (n = 192)
District, n (%)			
Mocuba	378 (52%)	281 (52%)	97 (51%)
Quelimane	353 (48%)	258 (48%)	95 (49%)
Area, n (%)			
Rural	187 (26%)	137 (25%)	50 (26%)
Urban	544 (74%)	402 (75%)	142 (74%)
Study population group, n (%)			
CHWs/volunteers	165 (23%)	165 (31%)	0
PWH	186 (25%)	186 (35%)	0
Taxi drivers	188 (26%)	188 (35%)	0
Adults 18–49 years of age	76 (10%)	0	76 (40%)
Adults 50+ years of age	77 (11%)	0	77 (40%)
Community/faith leaders	39 (5%)	0	39 (20%)
Sex, n (%)			
Female	345 (47%)	260 (48%)	85 (44%)
Male	386 (53%)	279 (52%)	107 (56%)
Age, years (n = 6 missing)			
Mean (SD)	36 (13)	32 (10)	46 (17)
Median (IQR)	32 (25–45)	30 (24–37)	50 (32–58)
Age, years (categorized) (n = 6 missing), n (%)			
18–24 years	169 (23%)	137 (26%)	32 (17%)
25–34 years	234 (32%)	212 (40%)	22 (12%)
35–49 years	185 (26%)	148 (28%)	37 (20%)
50+ years	137 (19%)	39 (7%)	98 (52%)
Educational level (n = 2 missing), n (%)			
No formal education/incomplete primary	192 (26%)	128 (24%)	64 (33%)
Primary (7th grade)	217 (30%)	161 (30%)	56 (29%)
Secondary (10th grade)	136 (19%)	110 (20%)	26 (14%)
Pre-university (12th grade)	165 (23%)	129 (24%)	36 (19%)
Superior/university	12 (2%)	3 (1%)	9 (5%)
Technical professional	7 (1%)	6 (1%)	1 (1%)
Marital status (n = 3 missing), n (%)			
Divorced/separated/widow	472 (65%)	80 (15%)	39 (20%)
Married/living together	137 (19%)	355 (66%)	117 (61%)
Single (not living with partner)	119 (16%)	101 (19%)	36 (19%)
Income status, n (%)			
Has any income	584 (80%)	478 (89%)	106 (55%)
Has no income	147 (20%)	61 (11%)	86 (45%)
Mother language (n = 4 missing), n (%)			
Local language	603 (83%)	476 (89%)	127 (66%)
Portuguese	124 (17%)	59 (11%)	65 (34%)
Number of household members, n (%)			
1	12 (2%)	10 (2%)	2 (1%)
2–5	432 (59%)	332 (62%)	100 (52%)
6–9	250 (34%)	171 (32%)	79 (41%)
10+	37 (5%)	26 (5%)	11 (6%)

N = 731.

Abbreviations: CHW, community health worker; FGD, focus group discussion; IQR, interquartile range; PWH, persons with human immunodeficiency virus; SD, standard deviation.

### Knowledge of COVID-19 and COVID-19 Vaccination

Among the 539 participants surveyed, 46% (247) felt they had a lot or some knowledge of COVID-19 and more than half (314; 59%) believed that COVID-19 infection was severe and would require hospitalization. When comparing the proportion by region (rural or urban), a difference was seen regarding the perception of disease severity, where a greater proportion of participants from rural areas (97; 72%) felt the disease would be severe (needing hospitalization) than those living in urban areas (217; 55%) (Table 3). All FGD participants reported that they had already heard about COVID-19 and felt that COVID-19 was a severe and dangerous disease that could quickly lead to death after becoming infected.

“…a disease that emerged in 2019 in China and spread throughout the world, ah Mozambique, it entered Mozambique in 2019 (or) 2020 and it is expanding, […] I mean it is spreading, it is killing many people and a lot of people are also getting infected with this disease, we are all afraid of this disease, it is a deadly disease, anyone is afraid of it, […] so it really is a disease that we know and are very afraid of….” (Community leader, Quelimane District, urban)

“…yes, it is also worth mentioning what I have heard that it really is a very dangerous disease, it kills in less time….” (Male adult, 18–49 years of age, Mocuba District, urban)

**Table 3.** Knowledge About COVID-19 and Information on COVID-19 Vaccination Among Survey Respondents, by Type of Area (Urban/ Rural)

	Total (n = 539)	Rural (n = 137)	Urban (n = 402)	*P*
How do you classify your knowledge about COVID-19? (n = 2 missing), n (%)				.62
None	18 (3.4%)	5 (3.7%)	13 (3.2%)	
A little	272 (50.7%)	71 (52.2%)	201 (50.1%)	
Some	213 (39.6%)	49 (36.0%)	164 (40.9%)	
A lot	34 (6.3%)	11 (8.1%)	23 (5.8%)	
How serious do you think COVID-19 can be (how many people will feel sick)? (n = 6 missing), n (%)				.001
Most will not have any symptoms	51 (9.6%)	4 (3.0%)	47 (11.8%)	
You can get sick but you will not need to be hospitalized	151 (28.3%)	32 (23.7%)	119 (29.9%)	
Many people will be very sick and will need to be hospitalized	314 (58.9%)	97 (71.9%)	217 (54.5%)	
Don't know	17 (3.2%)	2 (1.4%)	15 (3.8%)	
Do you think there is treatment for COVID-19? (n = 7 missing), n (%)				.77
Yes	205 (38.5%)	53 (39.0%)	152 (38.4%)	
No	198 (37.2%)	53 (39.0%)	145 (36.6%)	
Not sure/don't know	129 (24.3%)	30 (22.0%)	99 (25.0%)	
How can you prevent COVID-19 infection?,^a^ n (%)				
Washing hands	517 (95.9%)	131 (95.6%)	386 (96.0%)	1
Using facemask	492 (91.3%)	123 (89.8%)	369 (91.8%)	.59
Social distance	461 (85.5%)	113 (82.5%)	348 (86.6%)	.30
Use disinfectant	216 (40.1%)	24 (17.5%)	192 (47.8%)	<.001
Covering nose/mouth	128 (23.7%)	40 (29.2%)	88 (21.9%)	.11
Vaccination	35 (6.5%)	4 (2.9%)	31 (7.7%)	.08
Traditional medicine	15 (2.8%)	0 (0.0%)	15 (3.7%)	.016
Antibiotics	2 (0.4%)	0 (0.0%)	2 (0.5%)	1
Have you heard of COVID-19 vaccination? (n = 2 missing), n (%)				
Yes	534 (99.4%)	134 (97.8%)	400 (100.0%)	.016
No	3 (0.6%)	3 (2.2%)	0	
Where did you hear about the vaccine?,^a^ n (%)				
TV at home/community	375 (69.6%)	56 (40.9%)	319 (79.4%)	<.001
Radio	365 (67.7%)	85 (62.0%)	280 (69.7%)	.12
Conversation with healthcare workers	213 (39.5%)	49 (35.8%)	164 (40.8%)	.35
Conversation with family or friends	194 (36.0%)	42 (30.7%)	152 (37.8%)	.16
Information session at health facility	99 (18.4%)	37 (27.0%)	62 (15.4%)	.004
Social media	60 (11.1%)	7 (5.1%)	53 (13.2%)	.015
Leaflet	33 (6.1%)	7 (5.1%)	26 (6.5%)	.71
Newspaper	30 (5.6%)	7 (5.1%)	23 (5.7%)	.96
TV at health facility	23 (4.3%)	10 (7.3%)	13 (3.2%)	.07
Do you think the information was sufficient?, n (%)				.043
Yes	392 (73.5%)	108 (80.6%)	284 (71.2%)	
No	141 (26.5%)	26 (19.4%)	115 (28.8%)	
What source of information do you trust?,^a^ n (%)				
TV	351 (65.1%)	52 (38.0%)	299 (74.4%)	<.001
Radio	327 (60.7%)	77 (56.2%)	250 (62.2%)	.26
Conversation with healthcare workers	191 (35.4%)	31 (22.6%)	160 (39.8%)	<.001
Conversation with family or friends	94 (17.4%)	13 (9.5%)	81 (20.1%)	.007
Information session at HF	70 (13.0%)	34 (24.8%)	36 (9.0%)	<.001
Community leaders	34 (6.3%)	9 (6.6%)	25 (6.2%)	1
Newspaper	26 (4.8%)	7 (5.1%)	19 (4.7%)	1
Leaflet	26 (4.8%)	6 (4.4%)	20 (5.0%)	.96
Social media	27 (5.0%)	6 (4.4%)	21 (5.2%)	.87
TV at health facility	15 (2.8%)	7 (5.1%)	8 (2.0%)	.07
Community meetings	14 (2.6%)	0	14 (3.5%)	.03
Church	5 (0.9%)	3 (2.2%)	2 (0.5%)	.11

N = 539.

Abbreviations: COVID-19, coronavirus disease 2019; HF, health facility; TV, television.

^a^Participants were instructed to mark all that apply for these survey questions.

Almost all (534; 99%) had heard about COVID-19 vaccination: 98% in rural areas and 100% in urban areas (Table 3). Focus group analysis revealed that some participants agreed that a vaccine could prevent COVID-19 infection, although some were waiting to see if it could indeed prevent infection before they got vaccinated.

“I don't know if coronavirus has a cure, but I know we can prevent it through the vaccine.” (Male adult, 18–49 years of age, Quelimane District, urban)

“…now they said vaccines are coming, so that you can get vaccinated to see if this disease will end, so we are waiting for these vaccines to see if we don't get this disease, thank you.” (Male adult, 50+ years of age, Mocuba District, rural)

### Information and Information Sources on COVID-19 Vaccination

The most reported sources for information about the COVID-19 vaccine among the surveyed participants were television (TV) (70%; 375/539), radio (68%; 365/539), and conversations with healthcare workers (40%; 213/539). When comparing sources of information among respondents from rural and urban areas, in the rural areas 41% (56/137) reported having received information through television, 62% (85/137) through radio, and 36% (49/137) through conversations with healthcare workers compared with 79% (319/402), 70% (280/402), and 41% (164/402), respectively, in urban areas. These same 3 sources were most frequently reported as trustworthy. Almost 27% (141/539) found the information they received about COVID-19 to be insufficient (Table 3). Many community members mentioned in the FGDs that they heard about the existence of a COVID-19 vaccine via radio, television, and/or social media.

“I heard about it [the vaccine] on the television, on the radio, as well as in the neighborhoods, someone also passes by, some people also inform [about vaccination].” (Female adult, 50+ years of age, Quelimane District, urban)

“I got this information from the television hmm it's on the radio I could already hear it is our president day after day he always talks about it [the vaccine] yeah, I could already see it and also hear it on the radio….” (Male adult, 50+ years of age, Mocuba District, rural)

Despite mentioning various sources of information, participants felt that not all sources could be considered reliable. Survey respondents in both rural and urban areas most frequently reported that they trust TV and radio as sources of information (Table 3). Participants of the FGDs indicated that community leaders are the most trustworthy source of information, followed by health professionals, although they were less frequently mentioned than community leaders.

“They [community leaders] should be accompanied by health structure. These people are more suitable because they are the people who live with them daily and know what that person's behavior is, because a secretary, a zone chief, he knows that my community will receive this information in this and that way, because each community has its, its way of being or its way of receiving information, he could know how and when to talk about that information.” (Female adult, 18–49 years of age, Mocuba District, urban)

### Participants’ Vaccination Status

**Figure 2.**
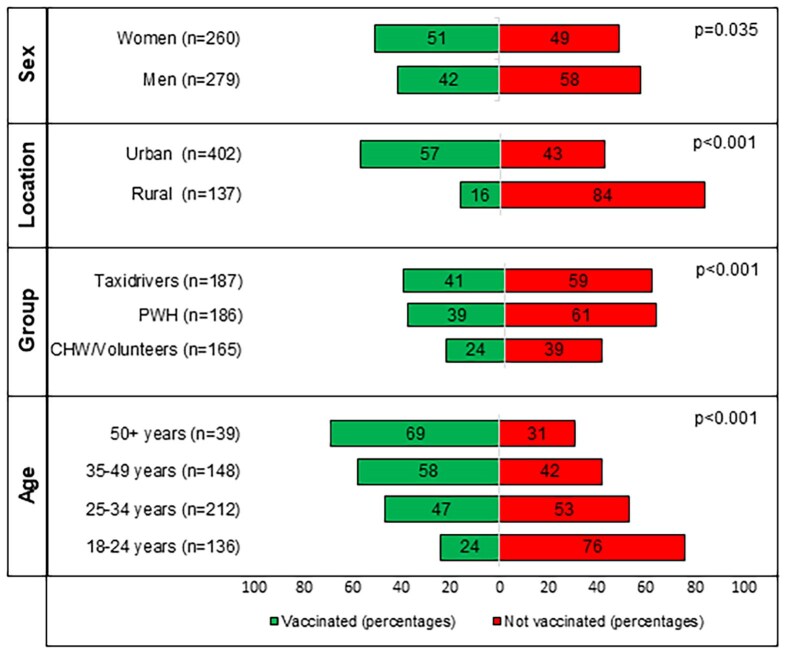
Acceptance of at least 1 dose of COVID-19 vaccination by sex, area, target group, and age group. Bivariate tests were performed using chi-square test. Abbreviations: CHW, community health workers; COVID-19, coronavirus disease 2019; PWH, persons with human immunodeficiency virus.

Nearly half (46%; 249/539) of survey respondents reported receiving at least 1 COVID-19 vaccine. More women (*P* = .04) and more persons in urban areas (*P* < .001) received at least 1 dose of the vaccine, and there was a reported 61%, 39%, and 41% uptake of at least 1 vaccine dose among the surveyed CHWs/volunteers, PWH, and taxi drivers, respectively (Figure 2). The multivariable GLMM, with age, sex, and target group being fixed effects and evaluation locations entered as random effects, showed that per 1-year increment in age, the odds of being vaccinated increased by 7% (adjusted odds ratio [aOR]: 1.07; 95% CI: 1.04–1.09; *P* < .001), while there was a trend seen of a lower odds of being vaccinated among men, but this was not significant (aOR: .61; 95% CI: .35–1.05; *P* = .08). Compared with CHWs/volunteers, PWH showed a significantly lower odds of being vaccinated (aOR: .35; 95% CI: .21–.57; *P* < .001), while taxi drivers had a nonsignificantly lower odds of being vaccinated (aOR: .79; 95% CI: .19–3.31; *P* = .75) (Table 4). Univariate analysis also showed that PWH who reported to have some level or a high level of knowledge about COVID-19 had higher odds of receiving at least 1 dose of vaccine compared with those reporting having no knowledge (OR: 4.31 [95% CI: 1.49–15.59; *P* = .012] and OR: 4.43 [95% CI: 1.29–18.26; *P* = .03], respectively).

**Table 4.** Multivariable Generalized Linear Mixed-Model of Receipt of COVID-19 Vaccine Among Survey Respondents

	aOR	95% CI	*P*
Fixed effects			
Age [per year]	1.08	1.05–1.10	<.001
Sex			
Female	Reference	…	
Male	.57	.33–1.00	.05
Group			
CHW/Volunteers	Reference	…	
PWH	.40	.24–.68	<.001
Taxi drivers	.95	.23–3.97	.94
Education	Reference	…	
No formal education/incomplete primary	1.35	.77–2.39	.30
Primary	1.91	1.00–3.63	.049
Secondary	2.01	1.09–3.71	.025

N = 538. Missing data from 1 respondent who reported there was no active vaccination campaign in their district (at the time of the study).

Abbreviations: aOR, adjusted odds ratio; CI, confidence interval; CHW, community health worker; COVID-19, coronavirus disease 2019; HIV, human immunodeficiency virus; PWH, persons with human immunodeficiency virus.

Among those who received at least 1 dose (n = 249), 231 (93%) were completely vaccinated (ie, having received all recommended doses of the vaccine). In the univariate analysis, no statistical difference between men and women was seen for the complete vaccination rate (94% women, 92% men; *P* = .78), while persons in urban areas had a higher vaccination completeness rate (68% rural, 96% urban; *P* < .001).

Community respondents mentioned in the FGDs that most would accept vaccination, although they felt that some may not, especially if they do not see a government official leading by example.

“I wouldn't accept it without seeing a leader, a leader, while his arm has already been vaccinated.” (Male adult, 18–49 years of age, Mocuba District, rural)

### Motivators for COVID-19 Vaccination

The majority of surveyed participants who received the vaccine said that the principal motivator was the belief that vaccines protect themselves (72%; 178/249), followed by the belief that vaccines protect their family (17%; 42/249) (Table 5). Additional analysis per target group showed similar principal motivators (Supplementary Table 2).

**Table 5.** Reasons to Accept or Not Accept COVID-19 Vaccination Among Survey Respondents, by Type of Area (Rural/ Urban)

	Total	Rural	Urban	*P*
Why did you receive COVID-19 vaccine? (n, %)	n = 249	n = 22	n = 227	.14
Will protect me	178 (71.5%)	20 (90.9%)	158 (69.6%)	
Belief it will protect my family	42 (16.9%)	0 (0.00%)	42 (18.5%)	
It is my right, am a person at risk	8 (3.2%)	1 (4.6%)	7 (3.1%)	
Want to go back to normal life	8 (3.2%)	0 (0.00%)	8 (3.5%)	
Healthcare worker told me to get	4 (1.6%)	0 (0.00%)	4 (1.8%)	
Other	9 (3.7%)	1 (4.5%)	8 (3.5%)	
Why did you not receive COVID-19 vaccine? (n, %)	n = 289	n = 114	n = 175	<.001
Not eligible (as perceived by participant)	35 (12.1%)	25 (21.9%)	10 (5.7%)	
Long lines/no time	32 (11.1%)	3 (2.6%)	29 (16.6%)	
Nobody offered	29 (10.0%)	16 (14.0%)	13 (7.4%)	
No information on campaign and its location	25 (8.6%)	17 (14.9%)	8 (4.6%)	
Lactating	21 (7.3%)	2 (1.7%)	19 (10.9%)	
Vaccines finished/campaign finished	20 (6.9%)	8 (7.0%)	12 (6.9%)	
Vaccination post is distant	19 (6.6%)	6 (5.3%)	13 (7.4%)	
Campaign did not arrive in our neighborhood	18 (6.2%)	16 (14.0%)	2 (1.1%)	
Absence	16 (5.5%)	0 (0.0%)	16 (9.1%)	
Sick	14 (4.8%)	0 (0.0%)	14 (8.0%)	
Fear to get the virus through vaccination	12 (4.2%)	4 (3.5%)	8 (4.6%)	
I don't believe it works/prevents	11 (3.8%)	7 (6.1%)	4 (2.3%)	
Don't belief save, don't trust, undecided	10 (3.5%)	2 (1.7%)	8 (4.6%)	
Pregnancy	10 (3.5%)	2 (1.7%)	8 (4.6%)	
Other	17 (5.9%)	6 (5.3%)	11 (6.3%)	

N = 538. Missing data from 1 respondent who reported there was no active vaccination campaign in their district (at the time of the study).

Abbreviation: COVID-19, coronavirus disease 2019.

Community members from both age groups (18–49 years and 50+ years) mentioned that some people are motivated to accept vaccination related to the fact that people who were vaccinated did not have any adverse reactions, and to the perceived risk of losing some rights such as being able to travel, accessing shops, markets, or banks if not vaccinated.

“I think the motivation is that the people who were vaccinated did not have any negative reactions, so from then on, people may have the desire to vaccinate too, because they didn't see someone saying that when they got the vaccine, they fell ill, ….” (Community leader, Mocuba District, rural)

“Others say, I'm going to get vaccinated because they give me that card, otherwise, I won't go into the bank or other service that I will need tomorrow until I get vaccinated.” (Community leader, Quelimane District, urban)

The need to see examples of people who were vaccinated and were doing well and to emulate people who serve as a reference were identified as facilitators for vaccination among the community members.

“I'm going to wait for [fictitious name] to go there and get vaccinated and see how he will be, so they've already seen 5, 10 people, but people are already healthy, he's already convinced to go and get vaccinated.” (Community leader, Quelimane District, urban)

### Barriers to COVID-19 Vaccination

When asking survey participants why he/she had not received their vaccination, the following major reasons were reported: the perception of not being eligible (12%; 35/289), lack of time/long lines (11%; 32/289), and not being offered the vaccine (10%; 29/289) (Table 5). Per target group, CHW/volunteers reported the perception of not being eligible, lactating, and not believing it works as a main barrier, while PWH reported lack of time/long lines, not having the campaign in the neighborhood, and taxi drivers reported lack of time/long lines, perception of not being eligible, and not being offered the vaccine as reasons not to be vaccinated (Supplementary Table 2).

As per the opinion of the community members, there were several reasons why they felt people did not adhere to vaccination recommendations. Myths/beliefs were the most frequently mentioned reasons not to vaccinate. The FGD participants felt that challenges related to access to information, such as lack of information, receiving the wrong information, and unanswered questions about the vaccine, did not favor/support vaccination uptake.

“I think they are denying it because of these comments that they are hearing that in 2 years they will lose their lives….” (Female adult, 18–49 years of age, Quelimane District, urban)

“It is difficult for people to go there without any explanation, without knowing why, … what are the advantages of the vaccine, what are the disadvantages, … we know that for everything there are advantages and disadvantages.…” (Female adult, 18–49 years of age, Mocuba District, urban)

“I also add the health technicians themselves sometimes when they are in the hospital … they say good things, but when they enter the community here with friends they also create demotivation … starts to reveal a little negative, … when they are there with their friends, at the bar, they say another thing, but when you are in the hospital, everything is fine.…” (Community leader, Quelimane District, urban)

Participants also mentioned, although less frequently, that some community members think the vaccine is a cure for COVID-19. Thus, they will only accept vaccination when they are infected.

“What is going through the minds of some brothers is the following: as the vaccine comes to cure, I haven't contracted COVID-19 yet, so I can't get vaccinated, why? … because the vaccine came to cure, it is one of the theories that people are using to avoid vaccination, they will only vaccinate when they are infected.…” (Male adult, 18–49 years of age, Quelimane District, urban)

Some FGD respondents—especially among the adult population—mentioned barriers regarding the vaccination: long lines at vaccination posts/delayed attendance, short periods for awareness campaigns in the more distant villages, long distances from the community to the vaccination post, and lack of clarity about priority target groups and vaccination points were also mentioned by participants as other factors that also contribute negatively to vaccination adherence.

### Communication Strategy

Participants in the FGDs suggested that information about COVID-19 vaccine and vaccination campaigns in rural areas should focus on communication talks led by health professionals in partnership with community leaders and/or CHWs/volunteers, as rural areas usually lack other means such as cellphone/smartphone, radio, or TV, which are used more in urban areas.

“For those who live here in the city, I believe you already have this information about the COVID-19 vaccine and also for those who live in remote areas, some have some, some don't, as in the case of those who have a cell phone, there are those who have a radio, but not everyone does. So, it would also be better if the group from the health unit, … to enter the hidden areas in person, because we can't just trust the transmission medium [social media], maybe not everyone heard it, so they could go there and expand the information in person with posters….” (Female adult, 18–49 years of age, Mocuba District, urban)

“What is very important is that in the absence of information bodies, because not everyone has devices to capture information, there is an organization that is responsible for spreading the benefits of these [types of] medical care and vaccines.” (Male adult, 50+ years of age, Quelimane District, urban)

Other proposals that the community members put forward included working with churches; having community activists, health staff, and local influencers (such as the community leaders) disseminate information in the communities; and conducting door-to-door campaigns to give clear and correct information regarding the vaccine.

“Also doing a door-to-door campaign would also be very good, as well as in neighborhood markets.” (Female adult, 18–49 years of age, Quelimane District, urban)

## DISCUSSION

This study is, to our knowledge, one of the first presenting results on the acceptance of COVID-19 vaccination during the vaccination campaigns in Mozambique. Our findings indicate a variable acceptance among adult populations residing in urban and rural areas within Zambézia Province, Mozambique.

Approximately half of the respondents of the survey reported having received at least 1 dose of the COVID-19 vaccine. While the adjusted odds of being vaccinated increased with older age, this may have been influenced by the age-related eligibility for vaccination at the time of the study. Acknowledging that not all of the interviewed persons were eligible at the time of the study, we did note that the eligible priority groups at the time of vaccination (CHWs/volunteers, taxi drivers, older adults aged 50+ years) did not have an optimal vaccine uptake. For 1 group (taxi drivers), the campaign had just started, which may explain the lower uptake. All CHWs/volunteers were eligible at the time of the study; however, uptake was lower than expected, possibly due to a lack of correct information regarding eligibility criteria for this group (eg, related to pregnancy and lactation). Among those who were not vaccinated, misinformation, especially regarding eligibility, was the main reason to refrain from vaccination. Vaccine uptake varies from context to context. A review focused on COVID-19 vaccine hesitancy in Africa conducted by Ackah et al [14] found that the rate of acceptance of the COVID-19 vaccine ranged from 6.9% to 97.9%. Being male, having a higher level of education, and fear of COVID-19 were the most reported factors associated with increased acceptance of the COVID-19 vaccine, while misinformation and concerns of vaccine safety resulted in hesitancy [14].

In the initial phase of vaccine roll-out, the World Health Organization (WHO) recommended using mass media to create demand and acceptance of COVID-19 vaccines [31, 32]. It endorsed delivering focused messages about vaccines and eligible populations via radio, TV, and other channels. The WHO guidelines also advocated that countries regularly track social and mainstream media to quickly identify misinformation and disinformation and provide real-time countermeasures to mitigate rumors [33]. A web-based cross-sectional study conducted in 2021, with 2572 participants of SSA origin, living in or outside of Africa, examined the impact of information sources on COVID-19 vaccine hesitancy and resistance in SSA. The study reported that people receiving information through TV, social media, healthcare staff, and family/friends were more likely to resist receipt of the COVID-19 vaccination [34]. In our study, we found that TV and radio were reported as the most frequently used sources of information and also the most reliable. Although not specifically analyzed in this evaluation, the authors note that it would be interesting to assess if the medium of communication influenced vaccine acceptance. In rural areas, healthcare workers and local leaders are perceived as being the best persons to be at the forefront of awareness campaigns. As access to TV or radio is not universal, the authors believe that, in rural areas, communication strategies, such as door-to-door campaigns, personal contact, and including local influencers in the campaigns, could be efficient ways to reach all communities. Additionally, we hypothesize that language could be a barrier in rural areas (in our study, only 17% reported Portuguese as their maternal language) as most nationally launched radio and TV spots were in Portuguese, therefore emphasizing the importance of including direct community talks that are performed in the local language.

Historically, many African countries lack immunization experience among adults or young people [8]. As vaccines are becoming more available for diseases such as cholera, Ebola, human papillomavirus (HPV), etc, it is essential to evaluate COVID-19 vaccination experiences to inform future vaccination campaigns, where community engagement and clear communication have been shown to be crucial for success [35, 36]. A survey conducted in Mozambique on hypothetical acceptance showed a variable acceptance over time, and demonstrated the importance of good and efficient communication strategies, as well as trust between the health sector and communities [37]. Approaches such as using “community champions,” volunteers trained to support vaccination campaigns in the communities, have been shown to play a critical role at promoting vaccine acceptance/uptake and could be adapted to other countries [38]. Experiences reported in South Korea found that the following factors played a role in the success of the COVID-19 vaccination campaigns of first and booster doses: pro-activeness, credibility, fighting misinformation, emphasizing social norms, and coherence [39]. Indeed, our study confirmed the importance of misinformation and strategies targeted to the local context in playing a role in increasing acceptability for first and likely for booster vaccine doses.

Despite the study being conducted in 2021, results can still provide valuable information on how to prepare for future public health emergencies. Lessons learned can contribute to vaccination strategies for adults (such as cholera vaccination) or for children, as reaching caregivers is essential (such as HPV vaccination). Our findings can also be used to understand rumors, as it has been shown that these can lead to misinformation and thus hesitancy [40]. Continuous health education may contribute to the acceptance and trust of health-related information on new vaccination campaigns, and may have a favorable impact on attitudes towards these public health strategies.

The study has several limitations: First, the study was conducted in only 2 districts in 1 province, and thus cannot be representative of the country. However, while our study was conducted during vaccination campaigns, studies on hypothetical acceptance (done before vaccination campaigns) in other regions in Mozambique had similar results in terms of reasons for nonacceptance, such as the belief that the vaccine doesn't work, or distrust [41, 42]. Second, there was a rapid increase in the availability of COVID-19 vaccines over a period of 6 months, with expansion of eligibility criteria for vaccination as per the national COVID-19 vaccination plan (ie, more groups became eligible in that time frame). Since the survey did not capture whether an individual respondent met the eligibility criteria for vaccination at the time of data collection, it was not possible for the study team to assess if a person was eligible or not at that time. Finally, there might have been selection bias, as only persons who were available on the day of data collection were invited to participate in the study, or social desirability response bias—the latter especially for community leaders who may be more likely to describe their response in more favorable terms. This risk was minimized by explaining before and during the discussions to speak freely, without limiting responses to the theoretical expected or preferred response.

### Conclusions

This study showed that information campaigns regarding COVID-19 vaccination reached the target groups, including in the more rural settings in Mozambique. Rural communities lean toward preferring community-based talks, with strong engagement/example from community leadership. Targeted campaigns are essential to attain desired coverage and to ensure all those in need of a vaccine are reached.

## Supplementary Material

ciaf054_Supplementary_Data

## Supplementary Data

Supplementary materials are available at *Clinical Infectious Diseases* online. Consisting of data provided by the authors to benefit the reader, the posted materials are not copyedited and are the sole responsibility of the authors, so questions or comments should be addressed to the corresponding author.

## Notes

***Author contributions.*** C. D. S., I. U. C., G. M., and C. L. conceptualized and designed the study. P. P. and F. P. coordinated the data collection. Z. Y. developed the quantitative data analysis plan and analyzed the data. C. L., A. Muteerwa, P. P., A. Manhiça, and I. U. C. analyzed the qualitative data. C. D. S., I. U. C., C. L., and A. Muteerwa synthesized and interpreted the results. L. N. and G. M. provided programmatic and contextual insights for understanding the results and guiding the discussion. I. U. C., C. D. S., and C. L. drafted initial and final drafts of the manuscript. All authors read, edited, and approved the final manuscript. C. W. W. and C. D. S. provided critical oversight and guidance throughout all stages of the evaluation.

***Acknowledgments.*** The authors thank all participants for their involvement in this evaluation and extend a special thank you to Erin Graves for her review of and contributions to the manuscript.

***Financial support.*** This work was supported by the Coronavirus Aid, Relief, and Economic Security (CARES) Act through the U.S. Centers for Disease Control and Prevention (CDC) Cooperative Agreement (U2GGH002367).

***Supplement sponsorship.*** This article appears as part of the supplement “COVID-19 Vaccine Research in Africa,” sponsored by the International Vaccine Institute and funded by the Coalition for Epidemic Preparedness Innovations (CEPI).

## References

[1] Wong MK, Brooks DJ, Ikejezie J, et al COVID-19 mortality and progress toward vaccinating older adults—World Health Organization, worldwide, 2020–2022. MMWR Morb Mortal Wkly Rep 2023; 72:113–8. 36730046 10.15585/mmwr.mm7205a1 PMC9927068

[2] World Health Organization (WHO) . WHO Coronavirus (COVID-19) Dashboard. Available at: https://covid19.who.int/. Accessed 28 December 2024.

[3] Msemburi W, Karlinsky A, Knutson V, Aleshin-Guendel S, Chatterji S, Wakefield J. The WHO estimates of excess mortality associated with the COVID-19 pandemic. Nature 2023; 613:130–7. 36517599 10.1038/s41586-022-05522-2 PMC9812776

[4] COVID-19 Excess Mortality Collaborators . Estimating excess mortality due to the COVID-19 pandemic: a systematic analysis of COVID-19-related mortality, 2020–21. Lancet 2022; 399:1513–36. 35279232 10.1016/S0140-6736(21)02796-3 PMC8912932

[5] World Health Organization (WHO) . WHO COVID-19 Dashboard—processed by Our World in Data. Available at: https://ourworldindata.org/coronavirus/country/mozambique#what-is-the-cumulative-number-of-confirmed-cases. Accessed 28 December 2024.

[6] Druedahl LC, Minssen T, Price WN. Collaboration in times of crisis: a study on COVID-19 vaccine R&D partnerships. Vaccine 2021; 39:6291–5. 34556366 10.1016/j.vaccine.2021.08.101 PMC8410639

[7] Watson OJ, Barnsley G, Toor J, Hogan AB, Winskill P, Ghani AC. Global impact of the first year of COVID-19 vaccination: a mathematical modelling study. Lancet Infect Dis 2022; 22:1293–302. 35753318 10.1016/S1473-3099(22)00320-6 PMC9225255

[8] Haddison EC, Machingaidze S, Wiysonge CS, Hussey GD, Kagina BM. Mapping the evidence-base of adolescent and adult vaccination in Africa: a slow but growing trend. J Vaccines Immunol 2019; 5:11–17.

[9] COVAX . COVAX: Working for global equitable access to COVID-19 vaccines 2020. Available at: https://www.who.int/initiatives/act-accelerator/covax. Accessed 13 December 2023.

[10] Moshin S, Capitine I. SARS-CoV-2 infection in Mozambique: epidemiology and advances made with vaccination against COVID-19. Anais Instituto de Higiene e Medicina Tropical 2022; 21:90–8.

[11] Ministry of Health, National Directorate for Public Health . Plano Nacional de Vacinação Contra a COVID-19. **2021.** Available at: https://www.misau.gov.mz/index.php/covid-19-planos-nacionais-e-vacinacao. Accessed 13 December 2023.

[12] MacDonald NE, SAGE Working Group on Vaccine Hesitancy. Vaccine hesitancy: definition, scope and determinants. Vaccine 2015; 33:4161–4. 25896383 10.1016/j.vaccine.2015.04.036

[13] Kadkhoda K . Herd immunity to COVID-19. Am J Clin Pathol 2021; 155:471–2. 33399182 10.1093/ajcp/aqaa272 PMC7929447

[14] Ackah BBB, Woo M, Stallwood L, et al COVID-19 vaccine hesitancy in Africa: a scoping review. Glob Health Res Policy 2022; 7:21. 35850783 10.1186/s41256-022-00255-1 PMC9294808

[15] Lazarus JV, Wyka K, White TM, et al A survey of COVID-19 vaccine acceptance across 23 countries in 2022. Nat Med 2023; 29:366–75. 36624316 10.1038/s41591-022-02185-4

[16] Miner CA, Timothy CG, Percy K, et al Acceptance of COVID-19 vaccine among sub-Saharan Africans (SSA): a comparative study of residents and diasporan dwellers. BMC Public Health 2023; 23:191. 36709269 10.1186/s12889-023-15116-w PMC9884132

[17] CDC-Africa . COVID-19 vaccination. Latest updates from Africa CDC on progress made in COVID-19 vaccinations on the continent. Available at: https://africacdc.org/covid-19-vaccination/. Accessed 27 October 2023.

[18] Instituto Nacional de Estatística . Population Census—Projections Population Zambézia 2017–2050. Available at: https://www.ine.gov.mz/web/guest/d/zambezia-1. Accessed 27 October 2023.

[19] Instituto Nacional de Saúde . Inquérito Sero-epidemiológico de SARS–CoV-2 (InCOVID-19), 2020. Relatório Final. Available at: https://ins.gov.mz/wp-content/uploads/2021/11/Relatorio-InCOVID-19-2020.pdf. Accessed 27 October 2023.

[20] Arnaldo P, Mabund N, Young PW, et al Prevalence of severe acute respiratory syndrome coronavirus 2 (SARS-CoV-2) antibodies in the Mozambican population: a cross-sectional serologic study in 3 cities, July-August 2020. Clin Infect Dis 2024; 75:S285–93. 10.1093/cid/ciac516 PMC9278262 35748663

[21] Momplaisir F, Haynes N, Nkwihoreze H, Nelson M, Werner RM, Jemmott J. Understanding drivers of coronavirus disease 2019 vaccine hesitancy among blacks. Clin Infect Dis 2021; 73:1784–9. 33560346 10.1093/cid/ciab102 PMC7929035

[22] Thanh Thi Le X, Ishizumi A, Thi Thu Nguyen H, et al Social and behavioral determinants of attitudes towards and practices of hepatitis B vaccine birth dose in Vietnam. Vaccine 2020; 38:8343–50. 33221065 10.1016/j.vaccine.2020.11.009 PMC10354407

[23] de Munter AC, Ruijs WLM, Ruiter RAC, et al Decision-making on maternal pertussis vaccination among women in a vaccine-hesitant religious group: stages and needs. PLoS One 2020; 15: e0242261. 33180859 10.1371/journal.pone.0242261 PMC7660565

[24] Hennink MM, Kaiser BN, Weber MB. What influences saturation? Estimating sample sizes in focus group research. Qual Health Res 2019; 29:1483–96. 30628545 10.1177/1049732318821692 PMC6635912

[25] Guest G, Bunce A, Johnson L. How many interviews are enough? An experiment with data saturation and variability. Field Methods 2006; 18:59–82.

[26] CDC-Africa . Majority of Africans would take a safe and effective COVID-19 vaccine. Available at: https://africacdc.org/news-item/majority-of-africans-would-take-a-safe-and-effective-covid-19-vaccine/. Accessed 16 March 2021.

[27] US Department of Health and Human Services (HSS) . 45 CFR part 46.101 (c). Available at: https://www.hhs.gov/ohrp/regulations-and-policy/regulations/45-cfr-46/index.html. Accessed 16 March 2021.

[28] US Department of Health and Human Services (HSS) . 21 CFR part 56. Available at: https://www.ecfr.gov/current/title-21/chapter-I/subchapter-A/part-56. Accessed 16 March 2021.

[29] R Core Team . R: a language and environment for statistical computing. Available at: https://www.R-project.org/. Accessed March 16th, 2021.

[30] Braun V, Clarke V. Using thematic analysis in psychology. Qual Res Psychol 2006; 3:77–101.

[31] World Health Organization . Acceptance and demand for COVID-19 vaccines: interim guidance. Available at: https://www.paho.org/en/documents/acceptance-and-demand-covid-19-vaccines-interim-guidance. Accessed 28 December 2024.

[32] World Health Organization . Guidance on developing a national deployment and vaccination plan for COVID-19 vaccines. Available at: https://www.who.int/publications/i/item/WHO-2019-nCoV-Vaccine-deployment-2021.1-eng. Accessed 28 December 2024.

[33] World Health Organization and United Nations Children's Fund (UNICEF) . Acceptance and demand for COVID-19 vaccines: interim guidance, 31 January 2021. Geneva, Switzerland: World Health Organization, 2021.

[34] Osuagwu UL, Mashige KP, Ovenseri-Ogbomo G, et al The impact of information sources on COVID-19 vaccine hesitancy and resistance in sub-Saharan Africa. BMC Public Health 2023; 23:38. 36609264 10.1186/s12889-022-14972-2 PMC9816548

[35] Collins J, Westerveld R, Nelson KA, et al Learn from the lessons and don't forget them: identifying transferable lessons for COVID-19 from meningitis A, yellow fever and Ebola virus disease vaccination campaigns. BMJ Glob Health 2021; 6:e006951. 10.1136/bmjgh-2021-006951 PMC8450956 34535490

[36] Amani A, Ngo Bama S, Dia M, et al Challenges, best practices, and lessons learned from oral cholera mass vaccination campaign in urban Cameroon during the COVID-19 era. Vaccine 2022; 40:6873–9. 36270892 10.1016/j.vaccine.2022.08.025 PMC9385732

[37] Hu B, Yang W, Bouanchaud P, et al Determinants of COVID-19 vaccine acceptance in Mozambique: the role of institutional trust. Vaccine 2023; 41:2846–52. 37003911 10.1016/j.vaccine.2023.03.053 PMC10040345

[38] Msunyaro E, Rangi J, Haonga T, et al Contribution of community champions to accelerate the uptake of COVID-19 vaccination in Rukwa region, Tanzania, February-October 2022. Pan Afr Med J 2023; 45(Suppl 1):5. 10.11604/pamj.supp.2023.45.1.39705 PMC10395113 37538368

[39] Hong SA . COVID-19 vaccine communication and advocacy strategy: a social marketing campaign for increasing COVID-19 vaccine uptake in South Korea. Humanit Soc Sci Commun 2023; 10:109. 36942012 10.1057/s41599-023-01593-2 PMC10018596

[40] Islam MS, Kamal AM, Kabir A, et al COVID-19 vaccine rumors and conspiracy theories: the need for cognitive inoculation against misinformation to improve vaccine adherence. PLoS One 2021; 16:e0251605. 33979412 10.1371/journal.pone.0251605 PMC8115834

[41] Dula J, Mulhanga A, Nhanombe A, et al COVID-19 vaccine acceptability and its determinants in Mozambique: an online survey. Vaccines (Basel) 2021; 9:828. 34451953 10.3390/vaccines9080828 PMC8402577

[42] Doctors with Africa CUAMM (Collegio Universitario Apiranti e Medici Missionari) . Mozambique: the desire to be vaccinated is strong. Available at: https://doctorswithafrica.org/en/whats-new/news/mozambique-the-desire-to-be-vaccinated-is-strong/. Accessed 28 December 2024.

