# Supplementary material for: Importance of Targeted Communication Strategies During COVID-19 Vaccination Campaigns in Mozambique: Results of a Mixed-Methods Acceptability Study
Source: Clin Infect Dis. 2025 Jul 22;80(Suppl 1):S66–77. doi: 10.1093/cid/ciaf054 (PMC12282518; doi:10.1093/cid/ciaf054)
Supplement: ciaf054_Supplementary_Data [file ciaf054_supplementary_data.zip › DeSchacht_COVID19_Vaccine_Acceptability_Supplementary_Table1_07FEB25.docx]

**Supplementary Table 1**. Summary of Target groups, per phase of COVID-19 vaccination, as per National COVID-19 Vaccination Plan.

| Target Group | | Criteria |
| --- | --- | --- |
| Phase 1 | | |
| 1 | Health care workers:  - National Health system  - Community Health worker  - Private Health sector | Health care workers are highly exposed to the infection. |
| 2 | Elderly living in elderly homes and staff at the elderly homes | Elderly represent the age group most at risk of for serious illness, hospitalization and death, which is why the occurrence of COVID-19 outbreaks in nursing homes nursing homes would result in high lethality in elderly residents. |
| 3 | Patients with Diabetes Mellitus (starting with those who are registered in the association of diabetes) | Diabetes represents a comorbidity of high risk for severe diseases, hospitalization and death. |
| 4 | Defense and Security forces | Defense forces (army) presents a high risk for infection due to difficulty in implementing prevention measures, and have a high mobility. |
| Phase 2 | | |
| 1 | Patients with Diabetes Mellitus not covered in phase 1 | Diabetes represents a comorbidity of high risk for severe diseases, hospitalization and death. |
| 2 | Prisoners and staff of prisons | In prisons, the risk of outbreaks of COVID-19 outbreaks is high due to confinement and the difficulty in implementing preventive measures. |
| 3 | 1. Patients on immunosuppressive therapy, patients with chronic renal on hemodialysis or on a waiting list 2. Patients with cardiac insufficiency or chronic respiratory insufficiency | Patients with comorbidities present a high risk for severe disease, hospitalization and death due to COVID-19 |
| 4 | Population aged 50 years or more in accommodation centers | - The risk of serious illness, hospitalization and death increases significantly after the age of 50 of age; - The implementation of prevention measures in accommodation centers is difficult due to the high crowding; - High mobility. |
| 5 | Population aged 50 years or more residing in urban areas | - The risk of serious illness, hospitalization and death increases significantly after the age of 50 of age; - Transmission of SARS CoV-2 in Mozambique is more intense in urban areas. |
| Phase 3 | | |
| 1 | Population aged 50 years or more in accommodation centers who were not covered in the previous phases | - The risk of serious illness, hospitalization and death increases significantly after the age of 50 of age; - The implementation of prevention measures in accommodation centers is difficult due to the high crowding; - High mobility. |
| 2 | Population aged 50 years or more residing in rural areas | The risk of serious illness, hospitalization and death  increases significantly after the age of 50  of age. |
| Phase 4 | | |
| 1 | All population not covered in the previous phases | Proportion towards a transition to a new normal is more secure through protection of the whole population. |
