# Supplementary material for: Importance of Targeted Communication Strategies During COVID-19 Vaccination Campaigns in Mozambique: Results of a Mixed-Methods Acceptability Study
Source: Clin Infect Dis. 2025 Jul 22;80(Suppl 1):S66–77. doi: 10.1093/cid/ciaf054 (PMC12282518; doi:10.1093/cid/ciaf054)
Supplement: ciaf054_Supplementary_Data [file ciaf054_supplementary_data.zip › DeSchacht_COVID19_Vaccine_Acceptability_Supplementary_Table2_07FEB25.docx]

**Supplementary Table 2.** Reasons to accept or not accept COVID-19 vaccination among survey respondents, by target group.

|  | **Total** | **CHW/Volunteers** | **PWH** | **Taxi Drivers** | **P** |
| --- | --- | --- | --- | --- | --- |
| Why did you accept to receive the COVID-19 vaccine? (n, %) | n=249 | n=101 | n=72 | n=76 | 0.04 |
| Will protect me | 178 (71.5%) | 79 (78.2%) | 49 (68.1%) | 50 (65.8%) |  |
| Belief it will protect my family | 42 (16.9%) | 10 (9.9%) | 14 (19.4%) | 18 (23.7%) |  |
| It is my right, am a person at risk | 8 (3.2%) | 3 (3.0%) | 3 (4.2%) | 2 (2.6%) |  |
| Want to go back to normal life | 8 (3.2%) | 1 (1.0%) | 2 (2.8%) | 5 (6.6%) |  |
| HCW told me to get | 4 (1.6%) | 4 (4.0%) | 0 (0.0%) | 0 (0.0%) |  |
| Other | 9 (3.6%) | 4 (4.0%) | 4 (5.6%) | 1 (1.3%) |  |
| Why did you not accept or receive the COVID-19 vaccine? (n, %) | n=289 | n=65 | n=114 | n=110 | <0.001 |
| Not eligible (as perceived by participant) | 35 (12.1%) | 13 (20.0%) | 8 (7.0%) | 14 (12.7%) |  |
| Long queue/ no time | 32 (11.1%) | 1 (1.5%) | 13 (11.4%) | 18 (16.4%) |  |
| Nobody offered | 29 (10.0%) | 2 (3.1%) | 9 (7.9%) | 18 (16.4%) |  |
| No information on campaign and its location | 25 (8.7%) | 7 (10.8%) | 9 (7.9%) | 9 (8.2%) |  |
| Lactating | 21 (7.3%) | 13 (20.0%) | 8 (7.0%) | 0 (0.0%) |  |
| Vaccines finished/ campaign finished | 20 (6.9%) | 5 (7.7%) | 10 (8.8%) | 5 (4.6%) |  |
| Vaccination post is distant | 19 (6.6%) | 4 (6.2%) | 6 (5.3%) | 9 (8.2%) |  |
| Campaign did not arrive in our neighborhood | 18 (6.2%) | 5 (7.7%) | 12 (10.5%) | 1 (0.9%) |  |
| Absence | 16 (5.5%) | 1 (1.5%) | 7 (6.1%) | 8 (7.3%) |  |
| Sick | 14 (4.8%) | 4 (6.2%) | 8 (7.0%) | 2 (1.8%) |  |
| Fear to get the virus through vaccination | 12 (4.2%) | 0 (0.0%) | 6 (5.3%) | 6 (5.5%) |  |
| I don’t believe it works/prevents | 11 (3.8%) | 7 (10.8%) | 3 (2.6%) | 0 (0.0%) |  |
| Don’t belief safe, don’t trust, undecided | 10 (3.5%) | 2 (3.1%) | 4 (3.5%) | 4 (3.6%) |  |
| Pregnancy | 10 (3.5%) | 1 (1.5%) | 3 (2.6%) | 7 (6.4%) |  |
| Other | 17 (5.9%) | 0 (0.0%) | 8 (7.0%) | 9 (8.2%) |  |

N=538. Missing data from 1 respondent who reported there was no active vaccination campaign in their district (at the time of the study)

CHW: Community Health Worker; PWH: Persons with HIV
